# Faster efficacy and reduced nodule occurrence with PLLA (poly-l-lactic acid) porous microspheres

**DOI:** 10.3389/fbioe.2025.1571820

**Published:** 2025-07-29

**Authors:** Qihua Cao, Jiayu Chen, Zijun Zhang, Yufei Xiong, Jinxia Ma, Wei Sun, Xin Chen, Qilei Lou, Kaijia Tang, Feng Lin, Yueliang Zhu, Xiaohua Yu

**Affiliations:** ^1^ Orthopedics Research Institute of Zhejiang University, Hangzhou, Zhejiang, China; ^2^ Key Laboratory of Motor System Disease Research and Precision Therapy of Zhejiang Province, Hangzhou, Zhejiang, China; ^3^ Clinical Research Center of Motor System Disease of Zhejiang Province, Hangzhou, China; ^4^ Zhejiang Chinese Medical University, Hangzhou, Zhejiang, China; ^5^ Hangzhou Philosopher’s Stone Biotechnology Co., Ltd., Hangzhou, Zhejiang, China

**Keywords:** porous microspheres, poly-l-lactic acid, injectable filler, prevention of nodules, collagen regeneration, aesthetic medicine

## Abstract

**Introduction:**

Poly-L-lactic acid (PLLA) has gained prominence as an injectable dermal filler as it can both stimulate collagen regeneration and deliver long-lasting effects. However, its application is often hampered by delayed therapeutic onset and adverse events, particularly nodule formation, likely due to uneven distribution caused by the easy formation of small clumps during PLLA reconstitution.

**Methods:**

In this study, PLLA porous microspheres were administered at six dorsal sites on one flank of New Zealand white rabbits, with six contralateral sites receiving Löviselle as control. At predetermined experimental time points, subjects were humanely euthanized. Post-euthanasia, tissue sections underwent histological examination through hematoxylin and eosin (H&E) staining and Masson’s trichrome technique, with subsequent statistical analysis of the observational data.

**Results:**

The PLLA porous microsphere group demonstrated significantly faster onset of action compared to the control, with observable collagen deposition as early as week 2, enhanced inflammatory cell infiltration, more homogeneous tissue distribution, and substantially fewer microaggregates upon histological examination.

**Discussion:**

Structural modification of PLLA microspheres has successfully accelerated their onset of action while reducing nodule formation, thereby providing novel insights for clinical applications.

## Introduction

As individuals age, a marked decline in skin elasticity and moisture levels occurs, leading to an increase in folds and wrinkles. This phenomenon is associated with epidermal thinning, dermal atrophy, a reduction in elastic tissue within the dermis, and a loss of dermal collagen. Injectable fillers have garnered increasing attention in the field of aesthetic medicine due to their simplicity and minimally invasive application. Injectable dermal fillers typically restore lost volume through two principal mechanisms: physical augmentation and stimulation of neocollagenesis (Guo et al., 2023). Physically augmenting fillers, such as hyaluronic acid (HA) and collagen, demonstrate immediate volumizing effects upon injection, though this effect gradually diminishes over time. In contrast, collagen-stimulating injectable fillers enhance long-term aesthetic outcomes by promoting collagen synthesis through the activation of macrophages and fibroblasts. These fillers commonly include materials such as polylactic acid (PLA), polycaprolactone (PCL), hydroxyapatite (CaHA), and polymethyl methacrylate (PMMA), which serve as the matrix for collagen deposition (Guo et al., 2023; Nowag et al., 2024; Eppley and Dadvand, 2006; Christen and Vercesi, 2020). However, a significant disadvantage is that the accumulation of these agents can lead to nodule formation or local calcification, posing risks of adverse effects.

Poly-L-lactic acid (PLLA) is a synthetic, biodegradable, and biocompatible polymer, approved by the FDA for use in various medical devices, including implants and injectable fillers. It is classified within the family of alpha-hydroxy acid polymers and has recently been utilized for the correction of fine wrinkles in the cheek area (Narins et al., 2010; Fabi et al., 2024). The mechanism of action of PLLA involves a regulated inflammatory response initiated by the immune system’s recognition of PLLA particles as foreign. This response initiates monocyte differentiation into macrophages, and the formation of foreign body giant cells. These cells then attract fibroblasts, which increase the levels of transforming growth factor-beta 1 (TGF-β1) and tissue inhibitors of metalloproteinases-1 (TIMP1), thereby promoting the synthesis of type I and type III collagen (Nowag et al., 2024; Sun et al., 2023; Oh et al., 2023; Bohnert et al., 2019). The first PLLA-based dermal filler Sculptra was approved by the FDA in 2004 and initially used to treat facial fat loss in HIV patients and later expanded its application to include the cosmetic correction of fine wrinkles in the cheek area, demonstrating broad clinical impact over time (Fabi et al., 2024; Carey et al., 2007; Moyle et al., 2004; Lam et al., 2006; Valantin et al., 2003). One of the most common adverse effects observed with Sculptra is the formation of subcutaneous papules and nodules, often due to factors such as improper reconstitution, uneven distribution of the product, superficial injection techniques, or insufficient post-treatment massage (Carey et al., 2007; Valantin et al., 2003; Signori, 2024; Vleggaar, 2006; Engelhard et al., 2005; Bachmann et al., 2009; Kadouch, 2017). Ensuring an even distribution of the PLLA product is crucial because technical challenges such as needle clogging, uneven dispersal, and micro-aggregation contribute to nodule formation (Bass, 2015; Narins, 2008; Lin et al., 2022; Vleggaar et al., 2014; Lin and Lin, 2021; Chen et al., 2020). To address these issues, research and development are focusing on the next-generation of PLLA fillers, incorporating porous microsphere technology to facilitate smoother injections and reduce the incidence of nodules.

**FIGURE 1:**
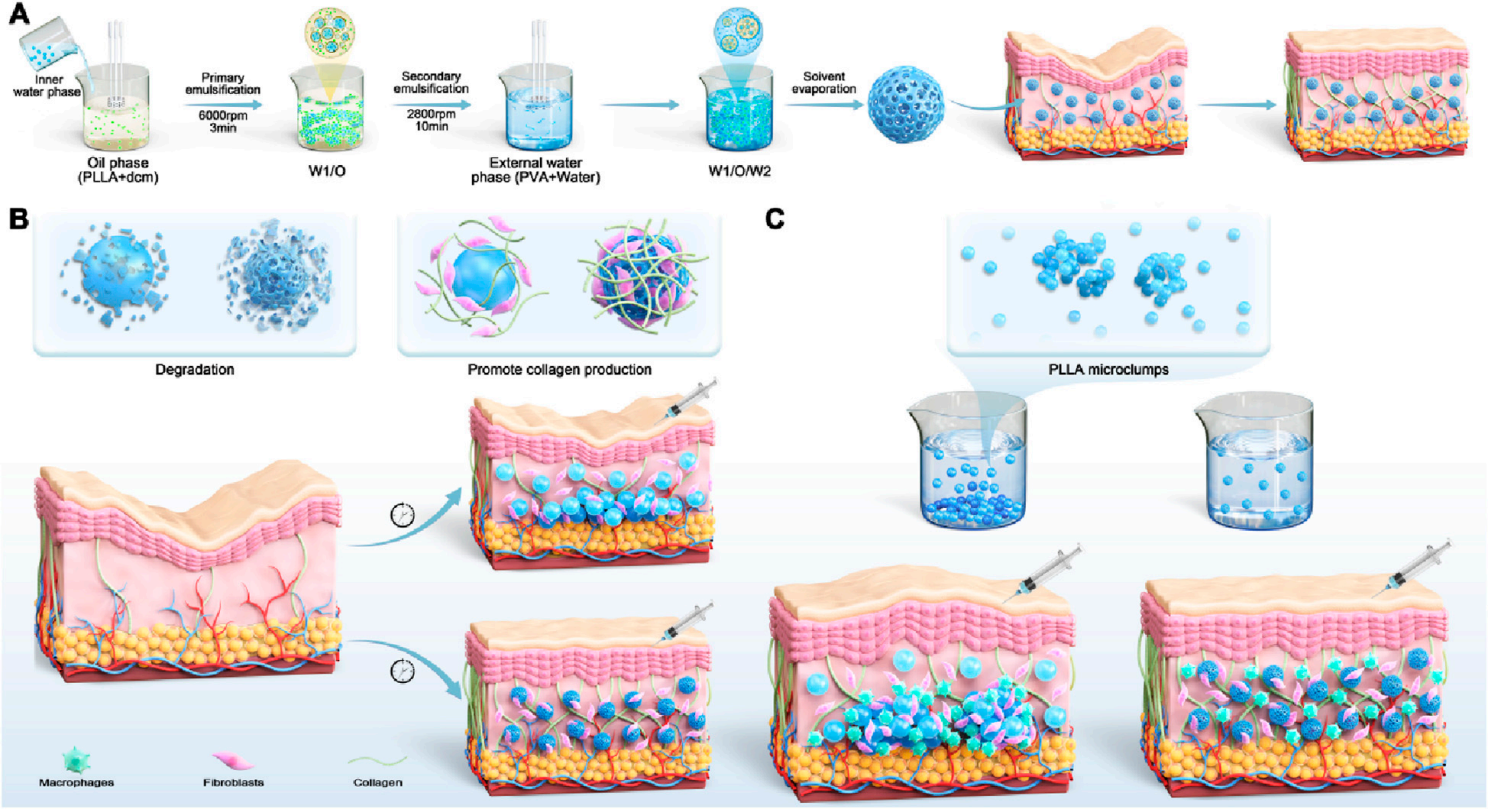
Schematic illustration of the fabrication and function of porous microspheres. **(A)** The PLLA porous microspheres are crafted using the double emulsion-solvent evaporation technique, which promotes collagen regeneration and combats skin sagging. **(B)** In comparison with solid microspheres, PLLA porous microspheres exhibit a quicker degradation rate and stimulate the production of increased amounts of collagen, rendering them more efficacious. **(C)** Compared to solid microspheres, PLLA porous microspheres exhibit a more uniform distribution upon reconstitution, which diminishes the aggregation of particles and thereby prevents the formation of nodules.

In this study, we fabricated porous PLLA microspheres via a double emulsion solvent evaporation method, employing NH_4_HCO_3_ as the pore-forming agent due to its efficiency in producing uniform and moderately sized pores. The distinguishing features of these porous microspheres lie in their reduced weight and increased surface area. Porosity levels were adjusted to investigate their impact on suspension stability, injectability, and biological activity. These attributes enable porous microspheres, in contrast to solid microspheres, to undergo degradation more rapidly, thereby initiating their functional effects earlier. Additionally, they allow the porous microspheres to remain suspended in water upon reconstitution, facilitating a more uniform distribution and mitigating the formation of nodules (Figure 1). Our experimental analysis revealed that microspheres with medium porosity not only maintained suspension up to 7 h post-reconstitution but also required lower injection forces similar to those in the low porosity group. Advanced imaging and *in vivo* staining, such as SEM, Hematoxylin and Eosin (HE), and Masson’s trichrome, indicated these medium porosity microspheres supported faster degradation, enhanced immune cell infiltration, and increased collagen production compared to their solid counterparts. Overall, optimization of porous PLLA microspheres via NH_4_HCO_3_ assisted double emulsion approach significantly improves their clinical application potential by reducing coagulation, promoting sustained product distribution, and accelerating effective outcomes in tissue integration and regeneration.

## Results

### Effect of different porogens on microsphere characteristics

**FIGURE 2:**
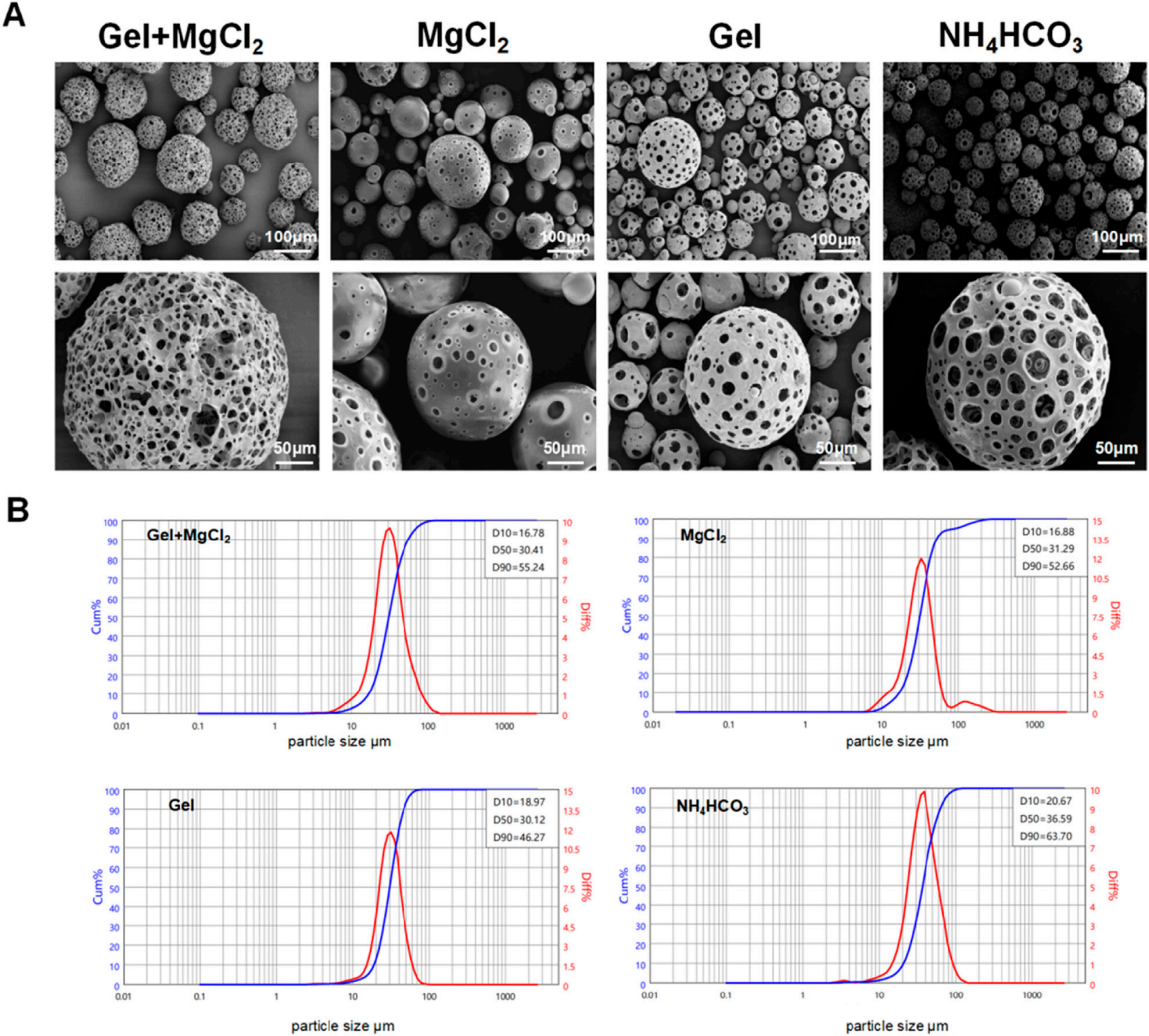
The influence of different pore forming agents on PLLA porous microspheres. **(A)** Surface morphology of the PLLA porous microspheres under different inner water phase, 6%Gel+30%MgCl_2_, 30%MgCl_2_, 6%Gel, 4%NH_4_HCO_3_. **(B)** Particle size distribution of the PLLA porous microspheres under different inner water phase, 6%Gel+30% MgCl_2_, 30%MgCl_2_, 6%Gel, 4%NH_4_HCO_3_.

We successfully developed porous PLLA microspheres using a double emulsion method to optimize their porosity, essential for enhancing their functionality in anti-aging applications. The porosity of these microspheres facilitated suspension in liquids, simplifying injection processes, and promoting greater cell attachment and collagen synthesis. Porous particles were fabricated via double emulsion solvent evaporation as shown in Figure 1A (Rosca et al., 2004; Kumar et al., 2022; Nishimura and Murakami, 2021; Li et al., 2024; Liu et al., 2005). To assess the impact of different pore-forming agents on porous PLLA microspheres, four agents were explored: a combination of 6% gelatin with 30% MgCl_2_, 30% MgCl_2_ alone, 6% gelatin alone, and 4% NH_4_HCO_3_. Each was incorporated into the internal aqueous phase, with an oil-water ratio set at 150:30 for the PLLA solution to internal aqueous phase solution volume. The morphological traits of these microspheres were then scrutinized using scanning electron microscopy (SEM). The analysis revealed that microspheres from the 6% Gelatin +30% MgCl_2_ group presented pores of varying sizes and irregular shapes, while those solely from the 30% MgCl_2_ exhibited consistently round pores but had a diminished rate of pore formation. In contrast, the 6% Gelatin group produced microspheres with high porosity, characterized by uniformly round pores, limited internal structure, and enlarged internal voids. Meanwhile, the 4% NH_4_HCO_3_ group’s microspheres were distinguished by their circular pores, consistent sizing, extensive porosity, and intricate internal honeycomb-like structure (Figure 2A).

Subsequent analysis focused on the diameter distribution of porous microspheres within various experimental groups, employing D10, D50, and D90 values to represent the particle size distribution. D10, D50, and D90 denote the percentile diameters where 10%, 50%, and 90% of particles in the population are smaller in size, respectively. In the 6% Gelatin +30% MgCl_2_ group, the D10, D50, and D90 values were recorded as 16.78 μm, 30.41 μm, and 55.24 μm, respectively. Comparatively, the 30% MgCl_2_ group showed slightly different distributions with D10, D50, and D90 values of 16.88 μm, 31.29 μm, and 52.66 μm, respectively. The 6% Gelatin group presented values of 18.97 μm, 30.12 μm, and 46.27 μm for D10, D50, and D90, respectively. Notably, the 4% NH_4_HCO_3_ group exhibited wider size distributions with D10, D50, and D90 values of 20.67 μm, 36.59 μm, and 63.70 μm, respectively, as illustrated in Figure 2B. Given its higher porosity, regular surface morphology, and distinctive internal honeycomb structure that enhances cell adhesion, NH_4_HCO_3_ was selected for further use as the optimal pore-forming agent in our ongoing experiments.

### Effect of different oil-water ratios on porosity and particle size

**FIGURE 3:**
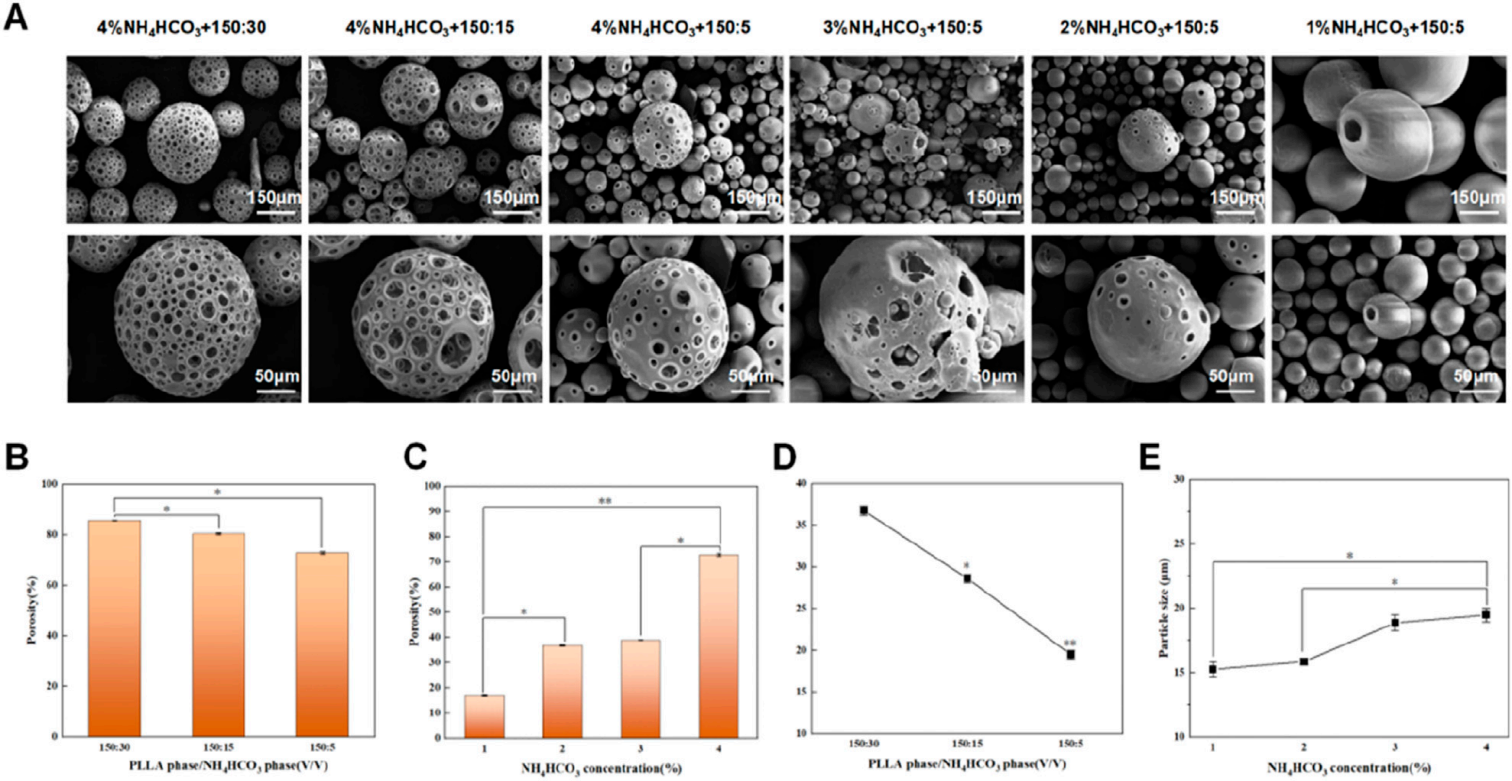
Evaluation of porous microspheres and regulation of porosity with NH4HCO3 as porogen. **(A)** Morphology of porous microspheres with different porosity. From left to right are 85.69%, 80.43%, 72.84%, 39.03%, 36.89%, 16.98%. **(B)** Effect of PLLA phase/NH4HCO3 phase on porosity (150:30, 150:15, 150:5). **(C)** Effect of NH4HCO3 concentration on porosity (4%, 3%, 2%, 1%). **(D)** Effect of PLLA phase/NH4HCO3 phase on particle size (150:30, 150:15, 150:5). **(E)** Effect of NH4HCO3 concentration on particle size (4%, 3%, 2%, 1%). The particle size refers to D50 values. Data are presented as mean ± SD (*n* = 3) (*p < 0.05; **p < 0.01).

The investigation into the effects of 4% NH4HCO3 as a pore-forming agent on the porosity and particle size of PLLA microspheres under varying oil-water ratios yielded insightful observations regarding the structural characteristics of the microspheres. SEM analysis was pivotal in revealing that an increase in the oil-water ratio correlated with a reduction in porosity. Lower porosity was found to potentially reduce bioactivity due to a decreased surface area for cell interaction. Despite this decrease, the microspheres maintained their distinctive internal honeycomb structure, which was crucial for their functionality in biomedical applications (Figure 3A). This structure was particularly valued for its potential to enhance interconnectivity within the spheres, facilitating better integration with biological tissues and improved delivery of therapeutic agent (Li et al., 2024; McCarthy et al., 2024; Yuan et al., 2021).

These results underscore the critical relationship between oil-water ratios and the structural characteristics of PLLA microspheres. The decreased porosity with higher oil concentrations suggests the agent cannot effectively induce porosity under these conditions. Meanwhile, the reduction in particle size associated with increasing oil-water ratios could be beneficial for applications requiring finer particles for improved dispersion and injectability in medical applications (Bentkover, 2009; Cohen et al., 2013; Alqahtani et al., 2020; Doshi and Mitragotri, 2010; Attenello and Maas, 2015; Lee and Kim, 2015; Herrmann et al., 2018). Thus, adjusting the oil-water ratio provides an adjustable processing parameter for customizing microsphere performance to optimizing the physical properties of PLLA microspheres for specific clinical uses.

### Effect of NH_4_HCO_3_ concentration on porosity and particle size

Next, we systematically analyzed the impact of varying NH_4_HCO_3_ concentrations (4%, 3%, 2%, 1%) on the porosity and particle size of porous microspheres while maintaining an oil-water ratio of 150:5. Utilizing scanning electron microscopy (SEM), we observed a significant reduction in porosity as NH_4_HCO_3_ concentration decreased (Figure 3A). Micropore formation on the surface was notably infrequent. The quantitative analysis showed a stark decrease in porosity from 72.84% at the highest concentration to 16.98% at the lowest (Figure 3C). Interestingly, the porosity levels between the 2% and 3% NH_4_HCO_3_ concentrations exhibited minimal variation, registering porosities of 36.89% and 39.03% respectively. In terms of particle size, an inverse relationship was observed with NH_4_HCO_3_ concentration; as the concentration increased, the median particle size (D50) also increased, ranging from 15.26 μm at 1% NH_4_HCO_3_ to 19.48 μm at 4% NH_4_HCO_3_ (Figure 3E). This correlation suggests that the concentration of NH_4_HCO_3_ not only influences porosity but also affects the overall dimensional growth of the microspheres, underscoring the critical role of NH_4_HCO_3_ in tuning both the structural and physical properties of these biomaterials for specific applications.

### Injectability and sedimentation rates of porous microspheres with different porosity

**FIGURE 4:**
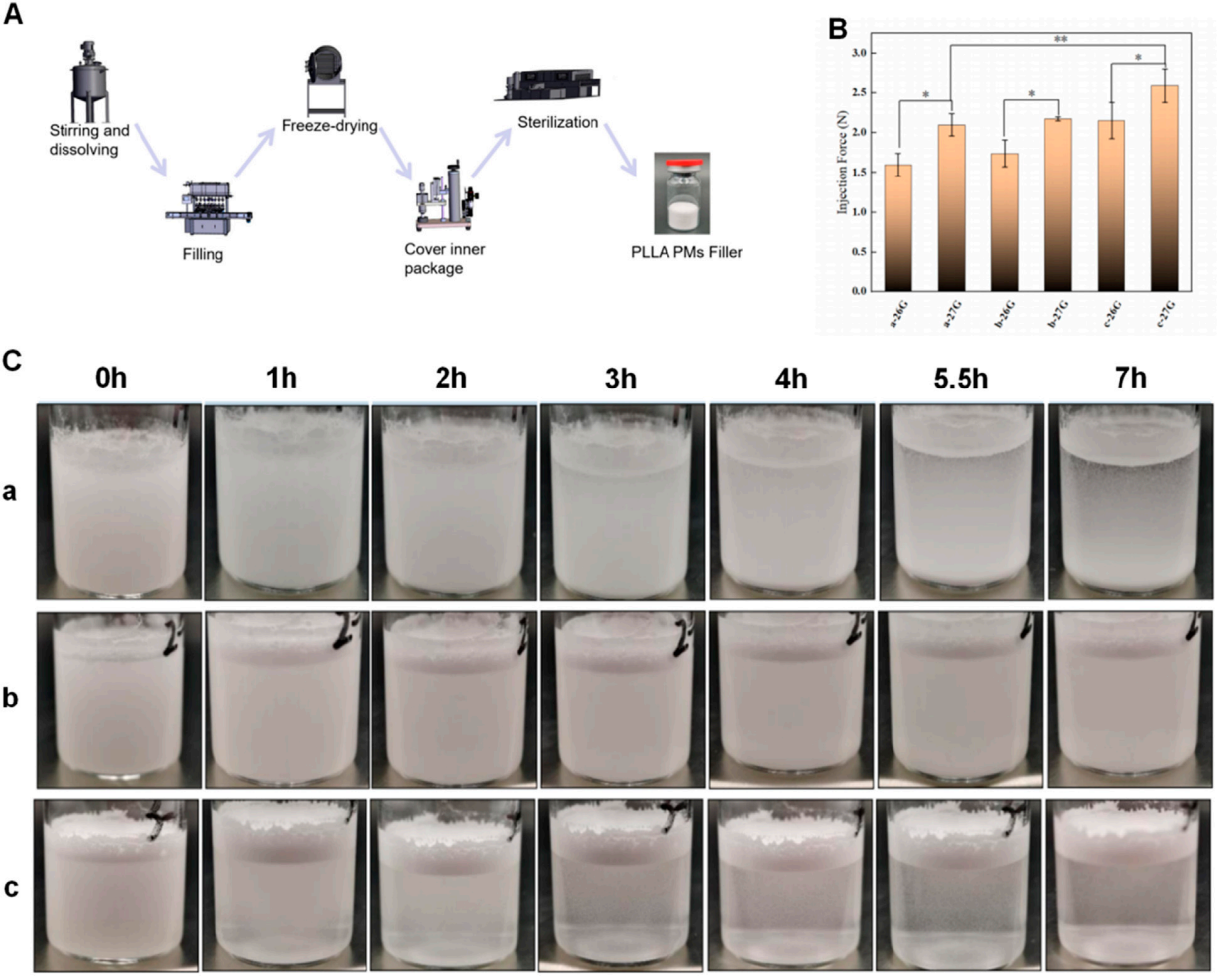
Performance evaluation of fillers prepared by PLLA porous microspheres with different porosity. **(A)** Schematic diagram showing the preparation of PLLA porous particle fillers. **(B)** Evaluation of injection properties of fillers with different porosity by two kinds of injection needles (26G, 27G). Data are presented as mean ± SD (*n* = 3) (*p < 0.05; **p < 0.01). **(C)** Suspension stability pictures during 7 h, a is low porosity group (<30%, 16.98%), b is medium porosity group (30%–60%, 39.03%), c is high porosity group (>60%, 72.84%).

Due to the importance of injectability and sedimentation rate of microspheres when used for dermal void fillers, we next explored the relationship between the porosity levels of microspheres synthesized with varying concentrations of NH_4_HCO_3_ (1%, 3%, and 4%) and their influences on the force required for administration. Utilizing an oil-water ratio of 150:5, the microspheres were categorized into three groups based on porosity: low (<30%, with actual porosity of 16.98%), medium (30%–60%, with actual porosity of 39.03%), and high (>60%, with actual porosity of 72.84%). Injection force experiments were conducted using 26G and 27G needles on reconstituted poly-L-lactic acid porous particles. Results indicated that injection forces for the 26G needle across the low, medium, and high porosity groups were 1.597 N, 1.740 N, and 2.151 N, respectively. For the 27G needle, forces were 2.098 N, 2.172 N, and 2.591 N, respectively (Figure 4A). Additionally, we evaluated the sedimentation behavior of these groups over 7 h post-reconstitution. The low porosity group demonstrated significant particle precipitation and minimal suspension, contrasting with the medium porosity group, which showed the highest level of suspended particles and moderate precipitation. The high porosity group displayed significantly reduced precipitation and suspended particles, with an increase in floating liquid (Figure 4B).

These findings demonstrated that microspheres with medium porosity not only required less injection force but also maintained a more stable suspension state post-reconstitution. This stability promoted homogeneous *in vivo* distribution of the microspheres upon injection, potentially enhancing the therapeutic efficacy of the delivered product (Zhao et al., 2023; Ginter et al., 2023). The results underscored the importance of optimizing microsphere porosity to balance ease of injection with effective distribution and stability in suspension, which were critical for achieving desired clinical outcomes in injectable application.

### Porous structure accelerated microspheres degradation and improved their performance *in vivo*

**FIGURE 5:**
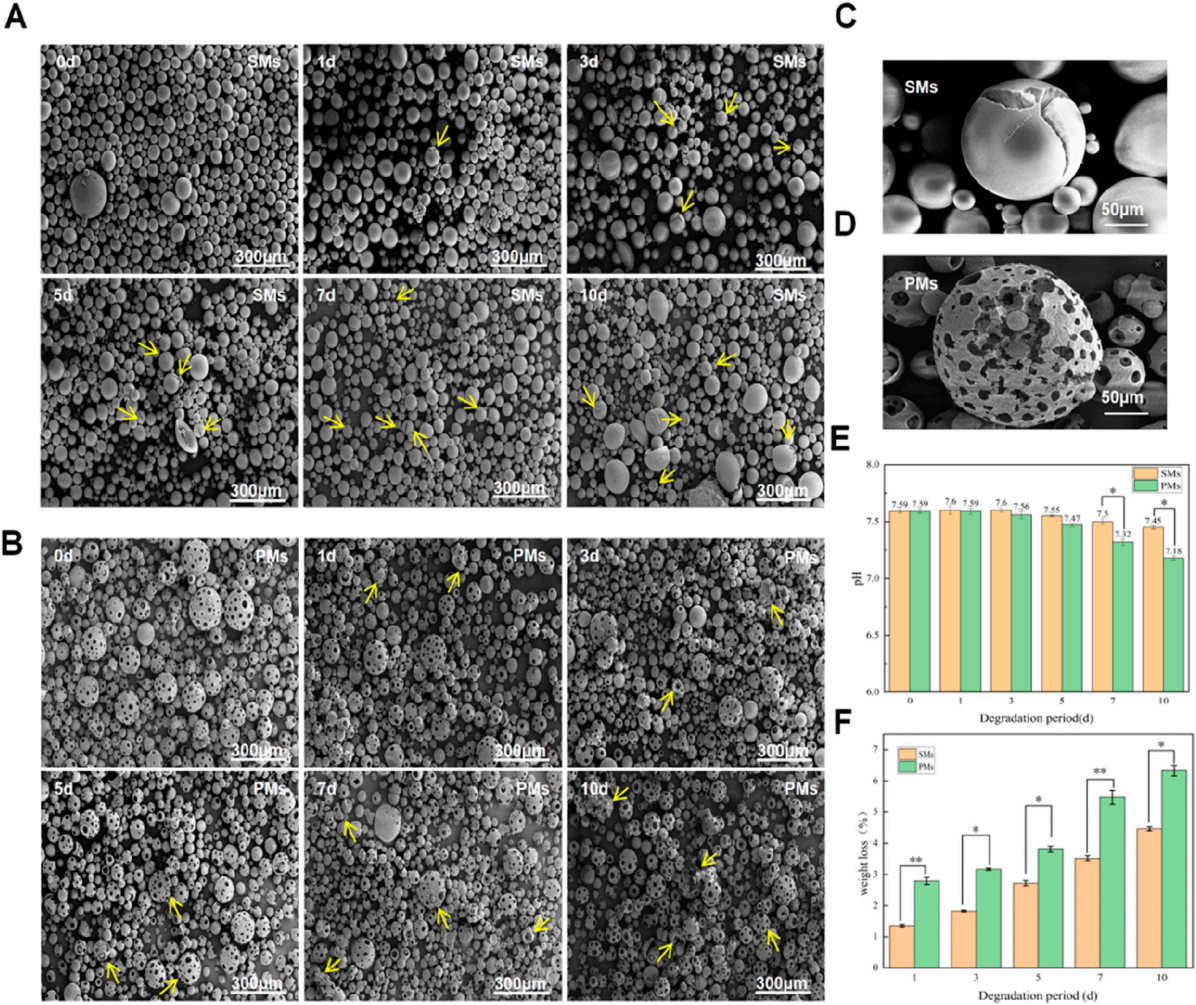
Evaluation of solid porous microspheres and porous microspheres *in vitro*. **(A)** SEM images of solid porous microspheres after different degradation periods (0, 1, 3, 5, 7, and 10 days), Yellow Arrow: solid porous microspheres with cracked surface. **(B)** SEM images of porous microspheres after different degradation periods (0, 1, 3, 5, 7, and 10 days), Yellow Arrow: the porous edge began to degrade until the porous microspheres collapsed. **(C)** Enlarged image of the cracking process of solid microspheres. **(D)** Enlarged image of the cracking process of porous microspheres **(E)** Weight loss of solid porous microspheres and porous microspheres after different time (1, 3, 5, 7, and 10 days). **(F)** pH values of solid porous microspheres and porous microspheres after different time (0, 1, 3, 5, 7, and 10 days). Data are presented as mean ± SD (*n* = 3) (*p < 0.05; **p < 0.01).

Next, we conducted microsphere degradation experiments to evaluate the influence of porous structure on the degradation behaviors of different microspheres: porous microspheres vs. solid microspheres. Both groups of microspheres were exposed to a soaking solution and incubated for varying durations (1, 3, 5, 7, and 10 days) to assess degradation rates at an elevated temperature to accelerate the degradation of PLLA. Degradation was quantitatively assessed through weight loss measurements and qualitatively through changes in surface integrity using Scanning Electron Microscopy (SEM). Solid microspheres exhibited microfractures visible via SEM, while porous microspheres degraded more uniformly, retaining their internal honeycomb structure even after the outer layer decomposed (Figures 5A–D). By day 10, weight loss was 4.46% for solid microspheres and 6.33% for porous ones. The pH of the soaking solution for solid microspheres was 7.45, compared to 7.18 for porous microspheres, indicating a more acidic environment due to faster degradation of the latter (Figures 5E,F). These findings demonstrate faster degradation kinetics in porous formulations and the porous microspheres are capable of preserving their internal honeycomb structure throughout the degradation process.

**FIGURE 6:**
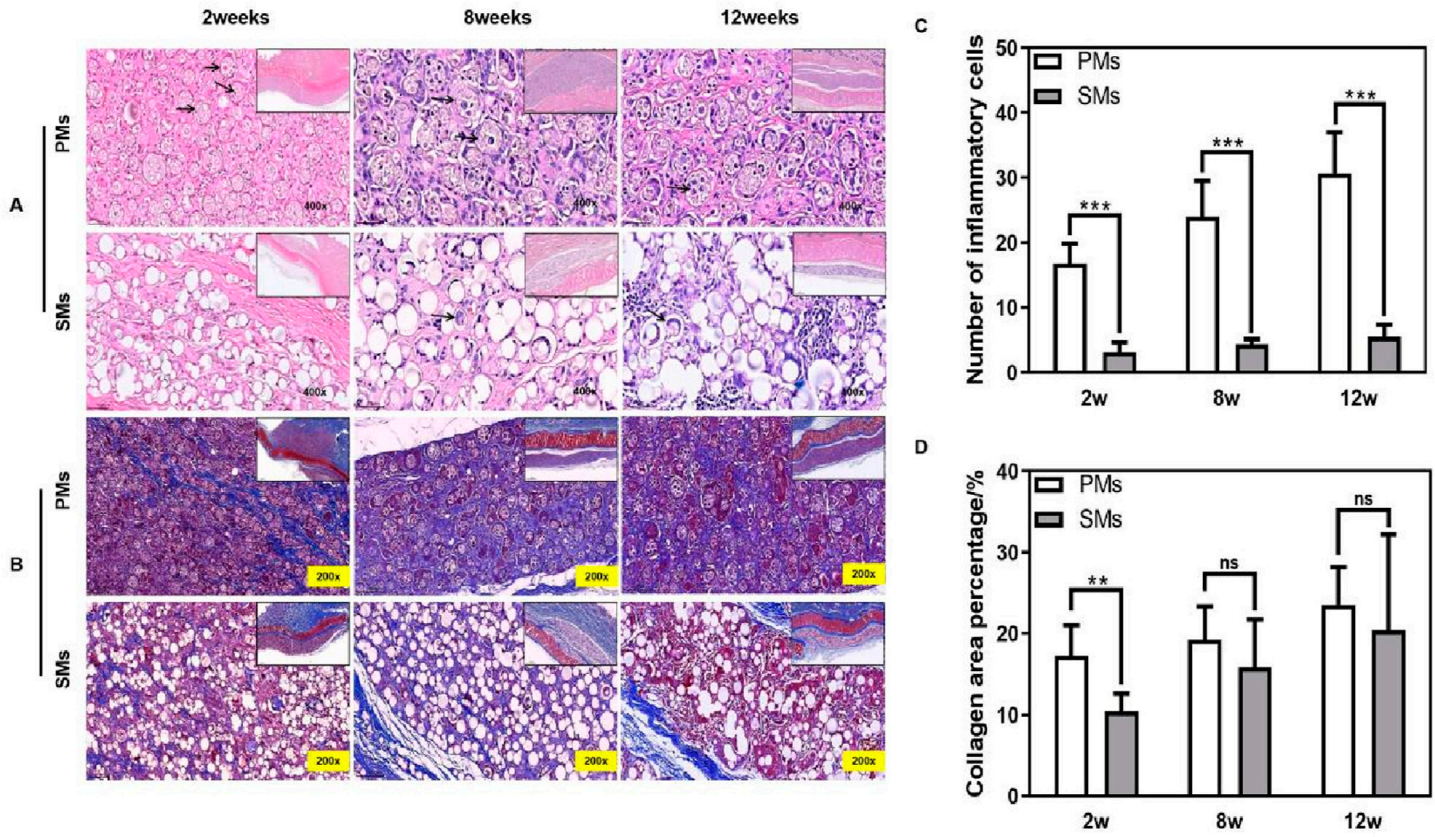
Solid microspheres vs. porous microspheres *in vivo*. **(A)** H&E staining (×400) at the 2nd, 8th and 12th week after injection. Black arrows: inflammatory cells, Solid boxes: zoom-in images of each sample. **(B)** Masson staining (×200) at the 2nd, 8th and 12th week after injection. Solid boxes: zoom-in images of each sample. **(C)** Histological evaluation of inflammatory cells. **(D)** Histological evaluation of collagen area ratio.

We employed a rabbit intradermal injection model to evaluate the *in vivo* performance of solid versus porous PLLA microspheres, assessing tissue response by administering medium-porosity porous PLLA microsphere fillers to the experimental group and treating the control group with LöviselleTM (a commercially available PLLA-based filler). Histopathological analysis revealed markedly enhanced inflammatory reactions in the porous microsphere group from week 2 through week 12, whereas the solid microsphere group exhibited only mild inflammation that became discernible at weeks 8 and 12 (Figure 6A). Quantitative histomorphometric analysis of inflammatory cell infiltration demonstrated significantly higher cell counts in porous microspheres throughout the observation period, reaching an approximate 30:5 ratio compared to solid microspheres by week 12 (Figure 6C). This pronounced inflammatory response may be attributed to synergistic effects of the porous architecture promoting cellular adhesion and accelerated degradation kinetics generating pro-inflammatory byproducts. Furthermore, robust collagen deposition was observed in porous microspheres at weeks 8 and 12, indicating enhanced extracellular matrix remodeling as evidenced by increased subdermal layer thickness (Figure 6B). Statistical evaluation of collagen area ratios revealed persistent yet temporally attenuated differences between groups, with porous microspheres showing a statistically significant 7% greater collagen deposition at week 2 (p < 0.05, Figure 6D), suggesting accelerated tissue regeneration kinetics. These comprehensive findings demonstrate that porous PLLA microspheres facilitate superior tissue integration through enhanced bioactivity and faster matrix deposition, establishing their potential as next-generation injectable dermal fillers with improved therapeutic efficacy.

### Porous microspheres significantly reduced nodule formation after implantation

**FIGURE 7:**
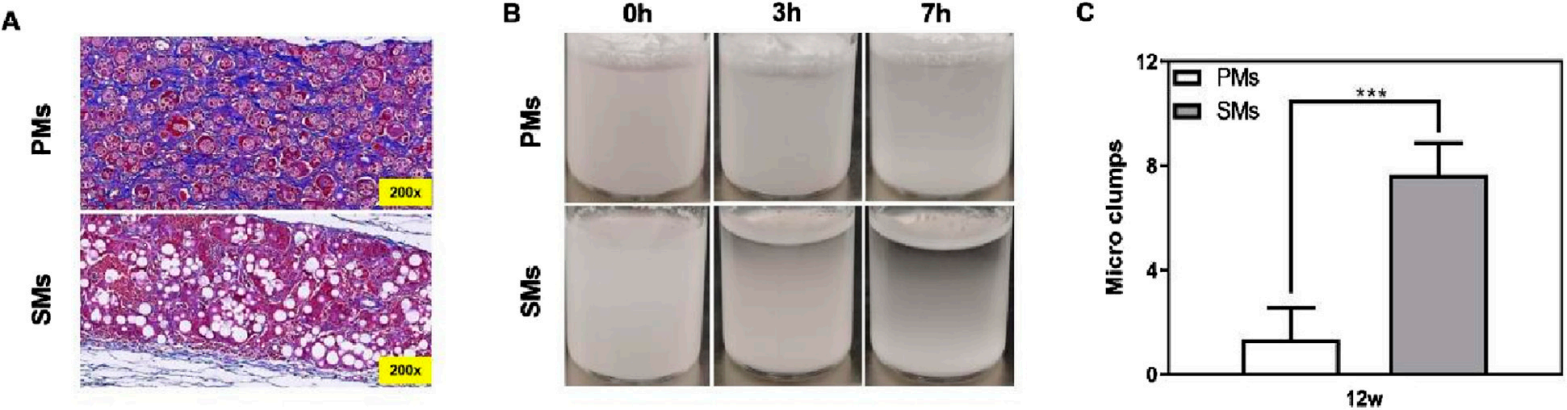
Injection of PLLA fillers may cause nodules *in vivo* and *in vitro* evaluation. **(A)** Masson staining (200×) is performed on the 12th week after subcutaneous implantation, the distribution of solid porous microspheres is disordered and shows aggregation and precipitation, while the distribution of porous microspheres is orderly and no aggregation and precipitation. **(B)** Evaluation of suspensibility of PLLA solid and porous microsphere fillers after redissolution. A large number of microspheres are precipitated in solid microspheres after 3 h, but the stability of porous microsphere is better in 7 h. **(C)** Histological evaluation of the number of micro clumps.

In clinical practice, the aggregation of solid microspheres, such as nodule formation, can result in localized inflammation and discomfort for patients. This aggregation can disrupt the uniform distribution of the therapeutic agents, leading to concentrated areas of high dosage that may not only decrease the efficacy of the treatment but also increase the risk of adverse reactions. In this set of experiments, we aimed to compare the performance and side effects of a commercial product, Löviselle™, with our optimized porous microspheres with proper porosity and good injectability. The objective was to evaluate the potential advantages of the porous microspheres in reducing side effects commonly associated with solid microsphere formulations, such as nodule formation and localized inflammation. Our results demonstrate that *in vivo*, porous microspheres achieve significantly more uniform subcutaneous distribution in New Zealand white rabbits compared to their solid counterparts, which exhibit a pronounced tendency toward aggregation and may consequently induce nodule formation. Quantitative analysis of microcluster formation further revealed a markedly lower incidence of small clusters in the porous microsphere group relative to the solid microsphere group, with a ratio of 7.67:1.33, underscoring the superior tissue distribution homogeneity of porous microspheres. Complementing these *in vivo* observations, *in vitro* experiments demonstrated that porous microspheres remained suspended significantly longer (7 h vs. 3 h; Figure 7B). These collective findings indicate that porous microspheres may confer substantial clinical advantages by ensuring more predictable therapeutic outcomes while simultaneously mitigating the potential for adverse effects.

## Discussion

We constructed porous poly (L-lactic acid) (PLLA) microspheres with an internal honeycomb-like structure in this study using a double-solvent evaporation method, employing ammonium bicarbonate (NH_4_HCO_3_) as a porogen. This design aimed to reduce the microspheres’ density while increasing their surface area, thereby reducing the lag phase for clinical efficacy. Additionally, the improved suspension stability upon reconstitution facilitates more uniform tissue distribution post-injection, minimizing nodule formation.

The selection of NH_4_HCO_3_ as a porogen was based on its ability to generate a homogeneous porous architecture, which not only enhances interfacial contact but also maintains structural integrity during degradation. Unlike alternative porogens such as sodium bicarbonate (NaHCO_3_) or calcium carbonate (CaCO_3_), NH_4_HCO_3_ decomposes entirely into gaseous byproducts (NH_3_, CO_2_, and H_2_O), eliminating risks associated with inorganic salt residues and pH fluctuations that could compromise product safety and performance (Sanchez-Herencia et al., 2021; Peng et al., 2017; Tewes et al., 2013).

Comparative evaluations of PLLA microspheres with varying porosities revealed that intermediate porosity (30%–60%) optimized both suspension duration and injection force (Figures 4B,C). While this range offers initial evidence, further refinement is required to determine the precise porosity threshold that maximizes clinical applicability.

*In vitro* and *in vivo* studies confirmed the accelerated efficacy of porous microspheres, though their long-term performance warrants careful evaluation. While collagen-mediated effects may mitigate potential disparities in prolonged outcomes, current animal data remain limited to a 12-week observation period, leaving long-term effects such as fibrotic remodeling or delayed immune reactions unexplored. Extended studies are necessary to assess degradation kinetics and therapeutic persistence beyond this timeframe.

Our research indicates that the degradation kinetics of PLLA microspheres are at a moderate level: they degrade faster than slowly degrading polycaprolactone (PCL, primarily degraded via ester hydrolysis with a degradation time of 2–4 years) but slower than cell-mediated calcium hydroxyapatite resorption (CaHA, typically 12–18 months). Currently, we have not conducted comparative studies between porous PLLA microspheres and other dermal fillers. We believe that performing such comparative studies will enable us to explore more potential application scenarios for porous PLLA microspheres.

Although PLLA porous microspheres accelerate the onset of action, a waiting period is still required to observe the full effect. To address this limitation, researchers have developed composite materials by combining PLLA with other substances. For instance, Su et al. synthesized a suspension of polylactic acid microspheres and hyaluronic acid (PLLA-b-PEG/HA), which demonstrated improved immediate filling effects while maintaining the long-term benefits of PLLA (Su et al., 2024). However, volume reduction after injection remains an issue due to the degradation of HA before PLLA achieves its complete efficacy.

Clinical studies have documented that the incidence of skin/subcutaneous nodules and granulomas, the most common adverse reactions associated with PLLA treatment, ranges from 1% to 44% (Ao et al., 2024). While current preventive measures primarily rely on refined injection techniques and post-treatment massage protocols, structural modifications of PLLA microspheres represent a novel approach to simultaneously address two key clinical challenges: accelerating the onset of therapeutic effects while reducing the incidence of nodules.

## Experimental section

### Preparation of porous microspheres

The PLLA, with an intrinsic viscosity of 1.8 dL/g, was sourced from Shenzhen Jusheng Biotechnology Co., Ltd. Porous microspheres were prepared using the double emulsion-solvent evaporation technique. Initially, the internal aqueous phase containing a pore-forming agent was introduced into the oil phase (composed of 150 mL of a 6% PLLA solution in dichloromethane) at a specific oil-to-water volume ratio. The biphasic mixture was then subjected to shear forces using a high-speed emulsifying shear machine at 6,000 rpm for 3 min to form a primary water-in-oil (w/o) emulsion. Subsequently, this primary emulsion was incorporated into an external aqueous phase of 1,500 mL of 1% polyvinyl alcohol (PVA), which had been pre-cooled to a temperature range of 2°C–8°C and sheared at 2,800 rpm for 10 min to form a complex water-in-oil-in-water (w/o/w) emulsion. The emulsion was then stirred in a fume hood using a stirrer at 100 rpm for 16 h (overnight). After allowing the mixture to stand for stratification, the obtained microspheres were washed twice with purified water at 40°C and subsequently washed twice with anhydrous ethanol. Finally, porous microspheres were dried in a forced-air oven at 40°C for 12 h.

### Scanning electron microscope

The microsphere sample was adhered on an aluminum substrate using carbon tape and sprayed a small amount of gold for 60 s. Each microsphere morphology and surface conditions were observed under scanning electron microscopy (SEM, Scios2Hivac) at an accelerating voltage of 3 kV.

### Particle diameter measurement

About 0.1 g of microspheres were weighed and thoroughly mixed with 10 mL of purified water. One to two drops of Triton X-100 solution were then added to create a homogeneous suspension. The laser diffraction particle size analyzer (Bettersize2600) was configured with an obscuration range set between 7% (upper limit) and 3% (lower limit), an ultrasonic frequency of 20 W, a rotational speed of 800 revolutions per minute, a sampling frequency of 7,200 times, a refractive index for the product particles of 1.451, and a refractive index for the dispersion medium of 1.333. The suspension was slowly introduced into the circulation cell and subjected to ultrasonic treatment for approximately 1 minute before measuring the D10, D50, and D90 particle size distributions.

### Determination of porosity

The formula for calculating porosity was given by Porosity = $\left(1-\frac{\rho_{0}}{\rho }\right)\times 100\%$ , where ρ denoted the density of PLLA solid microspheres and $\rho_{0}$ denoted the density of PLLA porous microspheres. The formula for calculating density was Density = $\frac{m-m_{0}}{5}$ , where m was the mass of a graduated cylinder filled with 5 mL of microspheres, and $m_{0}$ was the mass of the empty graduated cylinder. Through multiple measurements, the density of PLLA solid microspheres was found to be 0.79. Consequently, the porosity can be derived as Porosity = $\left(1-\frac{\rho_{0}}{0.79}\right)\times 100\%$ . By measuring the density of PLLA porous microspheres using the same method, the porosity of the PLLA porous microspheres can be obtained.

### Preparation of PLLA filler

In clinical applications, the PLLA injection filler often contains additives such as mannitol and sodium carboxymethyl cellulose. To minimize the influence of these additives on our experimental outcomes, we formulated a specific PLLA filler composition comprising 30 mg/mL of PLLA porous microspheres, 29 mg/mL of mannitol, and 9 mg/mL of sodium carboxymethyl cellulose in a 5 mL volume. This precisely formulated mixture was then dispensed into a vial, freeze-dried to remove moisture, and subsequently subjected to electron beam irradiation at a dose of 20 kGy for sterilization and stabilization purposes.

### Injection force test

The PLLA porous microspheres were formulated into a PLLA filler according to the aforementioned recipe. A 5 mL aliquot of the PLLA filler was reconstituted with 5 mL of purified water, followed by vigorous shaking for 1 min to ensure complete dissolution. The mixture was then allowed to stand for 2 h to promote stability. Subsequently, a 1 mL sample was aspirated using a syringe and placed into the mold of a servo tensile testing machine. The servo tensile testing machine was configured with a detection speed of 30 mm/min and a gauge length of 20 mm. The injection force was then measured and recorded for analysis.

### Static stratification experiment

The PLLA porous microspheres were formulated into a PLLA filler according to the previously established recipe. Each vial was filled with 5 mL of the PLLA filler, and subsequently, 5 mL of purified water was added. The mixture was vigorously shaken for 1 min and then allowed to stand. Photographic observations were conducted at specified time points according to the experimental setup.

### Accelerated degradation experiment

A soaking solution was prepared using 0.067 M potassium dihydrogen phosphate and 0.067 M disodium hydrogen phosphate, adjusted to maintain a pH of 7.4 ± 0.2. Porous microspheres were created using 3% NH_4_HCO_3_ as a pore-forming agent with an oil-to-water ratio of 150:5, achieving a porosity of 39.24%. For the control, a 6% (w/v) PLLA solution was emulsified into a 1% (w/v) PVA solution and subsequently solidified. Thirty centrifuge tubes were divided into five groups, each containing tubes loaded with 0.5 g of microspheres and 15 mL of the soaking solution, and were subjected to accelerated aging at 70°C ± 1°C for durations of 1, 3, 5, 7, and 10 days. Post-treatment, tubes were centrifuged, and the supernatant was analyzed for pH changes. Residues were dried to a constant weight for mass loss determination, providing a standardized method to assess material stability under accelerated conditions.

### Animal preparation

Four Male New Zealand white rabbits weighing 2–2.5 kg was provided by the Animal Experiment Center of Zhejiang University. Treatment of experimental animals was in accordance with the guidance of the Animal Care and Use Committee of the Medical College of Zhejiang University and all National Institutes of Health animal handling procedures (Approval number: AIRB-2023-0348). The animals had free access to both sterile water and food in a light and temperature-controlled environment. Rabbits were anesthetized by intramuscular injection of a mixture of zoletil (30 mg/kg) and rompun (10 mg/kg). Euthanasia was performed via intravenous administration of pentobarbital sodium at a dose of 100–200 mg/kg body weight.

### Histological analysis

The experimental group consisted of PLLA filler, which was synthesized using porous microspheres based on the previous formula. In contrast, the control group was administered Löviselle (Changchun Sheng Boma Biomaterials Co., LTD.). The dorsal fur of the white rabbits was carefully shaved, and subsequently, six experimental group samples were injected subcutaneously on one side, while six control group samples were injected subcutaneously on the opposite side, with each injection containing 0.2 mL. At the predetermined time for the experiment, the white rabbits were humanely euthanized. Following euthanasia, the specimens were dehydrated, embedded, and sectioned into 4 μm-thick paraffin slices. These slices were subsequently stained using Hematoxylin and Eosin (H&E) as well as Masson’s trichrome technique.

### Statistical analysis method

All data were presented as mean ± standard deviation. Statistical analyses were conducted using SPSS version 19.0 (IBM, United States) and involved univariate analysis of variance (ANOVA) followed by Tukey’s multiple comparison tests to evaluate inter-group differences. A P-value of less than 0.05 was considered statistically significant (*p < 0.05, **p < 0.01). Mapping was performed using Origin 2021 (OriginLab, United States).

## Data Availability

The original contributions presented in the study are included in the article/supplementary material, further inquiries can be directed to the corresponding authors.
